# Supplementary material for: Investigating Ethnic Disparity in Living-Donor Kidney Transplantation in the UK: Patient-Identified Reasons for Non-Donation among Family Members
Source: J Clin Med. 2020 Nov 21;9(11):3751. doi: 10.3390/jcm9113751 (PMC7700269; doi:10.3390/jcm9113751)
Supplement: Supplementary file 1 [file jcm-09-03751-s001.pdf]

Supplementary Materials

Study sites

The study was based at 14 hospitals in England and Northern Ireland: Belfast, Birmingham, Bristol, Cambridge, Epsom and St Helier, Guy's and St Thomas's, Imperial, Leicester, Manchester, Newcastle upon Tyne, Nottingham, Oxford, Sheffield, and St George's.

Interactions

In women there was no evidence of an association between Black, Asian and minority group ethnicity and reporting 'having no one to care for them after donation' as a reason for non-donation amongst their potential donors (unadjusted OR 1.42 [0.68-2.97]). However, in men there was evidence of a strong association between Black, Asian and minority group ethnicity and reporting potential donors (who may have been male or female) as not being able to donate due to not having someone to care for them after donation (likelihood of selecting reason for non-donation Black, Asian and minority group versus White unadjusted OR 5.76 [3.55-9.34]). Black, Asian and minority group women and White women were as likely to consider a lack of care post-donation as a reason their donors didn't donate, but Black, Asian and minority group men were more likely to report this as a potential barrier than White men.

Table S1. Responders and non-responders[a]

	**Total invited** n=3103 (%)	**Participants** n=1240 (%)	**Non-participants** n=1863 (%)
**Sex**			
Male	1902 (61)	705 (57)	1197 (64)
Female	1201 (39)	514 (41)	687 (37)
Missing	0	21 (2)	0
**Renal Transplant type**			
Live-donor	1462 (47)	672 (54)	790 (42)
Deceased-donor	1641 (53)	565 (46)	1076 (58)
Missing	0	3 (0.2)	0

[a]We could not analyse response by age as people were sampled by age at transplant and then asked to enter their current age in the questionnaire.

Table S2. Responders compared to national denominator population (data from NHS Blood and Transplant)

Characteristics		Study DDKT recipients<br><br>%	National population of DDKT recipients 2013-2017<br>%	Study LDKT recipients<br><br>%	National population of LDKT recipients 2013-2017<br>%
**Sex (%)**	**Male**<br>**Female**	58<br>42	62<br>38	58<br>42	59<br>41
**Age (years)**	**50-59**	28	26	27	23
	**60-69**	24	22	25	15
**Ethnicity (%)**	**White**<br>**Black, Asian and minority group /Other**	82<br>18	73<br>27	89<br>11	83<br>17

Individuals with missing data for each characteristic excluded from figures to allow direct comparison to published NHS Blood and Transplant data

Table S3. Missing data analysis

Variable	Missing data n (%)	Notes
Ethnicity	30 (2.4)	Those with missing data for age, ethnicity, and transplant type were more likely to have missing data for this variable. When focusing on individuals with recorded demographic data, no pattern of missingness with age (chi^2 p=0.73), or transplant type (chi^2 p=0.94), but maybe with sex (chi^2 p=0.03) – women were more likely to have missing data, but numbers were small. Only one person had missing ethnicity and education data.
Transplant type	3 (0.2)	Transplant type was missing as a result of the questionnaire code not being entered by participants who chose to complete the questionnaire online. Participants for whom transplant type was not recorded were more likely to also have missing data for age (2/3 had age missing, chi^2 p<0.001), sex (2/3 had sex missing, chi^2 p<0.001), and ethnicity (2/3 were missing ethnicity, chi^2 p<0.001).
Number of potential donors	50 (4.0)	50 participants had recorded no family members – this may mean that they have no living relatives or that the data is missing due to participants not wishing to complete this section. Record of this varied with age (chi^2 p<0.001): older participants age 60-69 years were more likely to have recorded no family members which might suggest that the data is not missing but that no relatives exist. Those with missing data for age, sex, ethnicity, and transplant type were also more likely to have missing data for this variable.<br>When focusing on individuals with recorded demographic data, no pattern of missingness with age (chi^2 p=0.17), sex (chi^2 p=0.89), but maybe with ethnicity (chi^2 p=0.001) – suggested that non-white ethnic patients' data more likely to be missing/blank and with transplant type (chi^2 p=0.003) suggesting recipients of deceased-donor kidney transplants were more likely to have missing data for potential donors. Again, as with age, this may suggest that the data is not missing but that no relatives exist.
Socioeconomic position: education	83 (6.7)	Those with missing data for age, sex, ethnicity, and transplant type were more likely to have missing data for this variable. When focusing on individuals with recorded demographic data (age, sex, ethnicity and transplant type), there was no pattern of missingness with age (chi^2 p=0.47), sex (chi^2 p=0.12), or transplant type (chi^2 p=0.17) but maybe with ethnicity (chi^2 p<0.001) – suggested that black patients' and those from 'other ethnic groups' are more likely to have missing data.
Age	34 (2.7)	Those with missing data for sex, ethnicity, and transplant type were more likely to have missing data for this variable. When focusing on individuals with recorded demographic data, no pattern of missingness was observed with sex (chi^2 p=0.05), education (no participants with age missing but education recorded), ethnicity (chi^2 p=0.97) or transplant type (chi^2 p=0.89).
Sex	21 (1.7)	Those with missing data for age, ethnicity, and transplant type were more likely to have missing data for this variable. When focusing on individuals with recorded demographic data, no pattern of missingness with age, ethnicity or transplant type (chi^2 p=0.75).

Table S4. Participant demographics – comparison of White respondents and Black, Asian and minority group respondents

	White participants (n= 1027) *n* (%)	Black, Asian and minority group[a] participants (n=171) *n* (%)	Chi²
**Sex**			
Female	438 (43)	64 (37)	**p=0.2**
Male	589 (57)	107 (63)	
**Type of transplant**			
Living-donor kidney transplant	581 (57)	72 (42)	**p<0.001**
Deceased-donor kidney transplant	445 (43)	99 (58)	
**Age group (years)[b]**			
20-29	56 (6)	18 (11)	
30-39	113 (11)	22 (13)	
40-49	180 (18)	27 (16)	**p=0.04**
50-59	275 (27)	53 (31)	
60-69	257 (25)	37 (22)	
70-79	134 (13)	13 (8)	
80-89	6 (0.6)	0	
**Religion**			
Christian	643 (65)	69 (43)	
Other religion (Muslim/Sikh/Hindu/Jewish)[c]	40 (4)	72 (45)	**p<0.001**
No religion	344 (31)	30 (12)	
**Number of potential donors** (median, IQR)	19 (16)	16 (11)	**p=0.02[d]**

[a]Black, Asian and minority group participants comprises UK ONS Ethnic Groups Asian/Asian-British, Black/African/Caribbean/Black British, Mixed/Multiple ethnic groups, Other Ethnic groups; [b]No participants aged <20 years; [c]Small numbers in some groups risked identification: we were therefore required to combine Muslim, Sikh, Hindu, Jewish respondents for analysis; [d]Wilcoxon rank-sum test

Table S5. Multivariable logistic regression analysis comparing reasons potential donor unsuitable between White and Black, Asian and minority group participants – missing explanatory variables imputed

Reason person unsuitable as donor	Black, Asian and minority group vs White Unadjusted OR [95% CI]	Black, Asian and minority group vs White Adjusted[a] OR [95% CI]
**Age – too old or too young**	1.01 [0.75-1.34]	1.00 [0.76-1.32]
**Health – not healthy enough**	1.03 [0.78-1.36]	1.02 [0.78-1.35]
**Weight – too over or underweight**	1.22 [0.83-1.77]	1.23 [0.84-1.80]
**Location – live too far away**	3.20 [2.21-4.62]	3.19 [2.20-4.63]
**Financial/cost – financial impact of donation would be too much**	2.87 [2.05-4.02]	2.90 [2.06-4.09]
**Job – no able to take time off work**	1.77 [1.15-2.73]	1.79 [1.16-2.77]
**Blood group – not the right blood group**	1.74 [1.39-2.17]	1.73 [1.39-2.16]
**No-one to care for them after donation**	3.55 [2.50-5.03]	3.50 [2.42-5.06]

[a]Adjusted for sex and age

Table S6. Characteristics of Black, Asian and minority group participants providing qualitative responses

	Participants (n= 56)<br><br>n (%)
**Sex**	
Female	22 (39)
Male	34 (61)
**Type of transplant**	
Living Donor Kidney Transplant	28 (50)
Deceased Donor Kidney Transplant	28 (50)
**Age group (years)**[a]	
20-29	4 (7)
30-39	7 (13)
40-49	3 (5)
50-59	21 (38)
60-69	15 (27)
70-79	5 (9)
Missing	1 (2)
**Ethnicity**	
Asian/Asian-British	30 (54)
Black/African/Caribbean/Black British	16 (29)
Mixed/Multiple ethnic groups	2 (4)
Other Ethnic groups	8 (14)
**Religion**	
Christian	20 (36)
Hindu	9 (16)
Sikh	8 (14)
Muslim	7 (13)
No religion	4 (7)
Other	3 (5)
Missing	5 (9)

[a]No participants aged <20 years

Table S7. Themes and illustrative quotes from thematic analysis of White participant responses

Theme	Representative quote
**Burden of disease within family**	"Suffer same genetic variation as me (aHUS)." (Male, 30-39 years, White, Christian, LDKT)<br><br>"All 5 relatives have the same condition as me. Polycystic kidney disease (PKD)." (Female 50-59 years, White, Christian, DDKT)<br><br>"Already had kidney transplant due to hereditary kidney disease." (Male 50-59 years, White, Christian, DDKT)<br><br>"Family history of kidney disease. 1. Member of family has had kidney transplant. 2. Daughter carries gene (genetic) FSGS." (Male 50-59 years, White, DDKT)<br><br>"1 son on dialysis has FSGS. 2nd son has same kidney disease FSGS. Sister has already given 1 of her kidneys to me." (Female 60-69 years, White, Christian, DDKT)<br><br>"Alcoholism, mental health etc." (Male, 20-29 years, White, Other, DDKT)<br><br>"Brother - liked to drink to[o] much and unreliable" (Male, 50-59 years, White, Christian, DDKT)
**Lack of close relationships**	"Don't know them well enough." (Male, 30-39 years, White, Christian, DDKT)<br><br>"The relationship isn't close enough to ask." (Male, 30-39 years, White, Jewish, LDKT)<br><br>"I sent an email to all my friends, telling them about my situation and that I was looking for a living donor. Some of them cut off all contact after that." (Female, 40-49 years, White, Christian, LDKT)<br><br>"They all could have donated. But the human brain is a pleasure seeking organ. And I think they (my family) would see me die rather than help. This was a big wake up call for me. As they are caring and Christian. This made me see truth, they way it is. It made me feel VERY alone." (Male 40-49 years, White, Other religion, DDKT)<br><br>"I have lost contact with all of my family since my parents divorce and I have no interest in any future contact." (Male 50-59 years, White, Other religion, DDKT)<br><br>"I come from a very dysfunctional family, split by my parents' divorce, and, further, by the fact that my mother has no relationship with her brother (my uncle). I sent an email to all my friends, telling them about my situation and that I was looking for a living donor. Some of them cut off all contact after that." (Female, 40-49 years, White, Christian, LDKT)
**Protecting others**	"I don't want to put their health at risk." (Male, 30-39 years, White, Christian, DDKT)<br><br>"My kids I would never take a kidney from." (Male 30-39 years, White, No religion, DDKT)<br><br>"Concern about impact on their children and family if things went wrong." (Male 50-59 years, White, Christian, DDKT)

	"One sibling offered and had tests but I asked them to decline as the only option was a triple swap i.e. they would give a kidney to a stranger and I would receive one from someone else. Essentially, this would have made them an altruistic donor. As they had a young family I felt this was a burden they should not have to bear. My first transplant was from another sibling which, unfortunately, never worked. I was not prepared to allow another sibling have a potentially unnecessary operation." (Female 50-59 years, White, Christian, DDKT)<br><br>"As I have a hereditary kidney condition which affects other family members there are some unaffected people who are siblings to affected members - I would not want them to put my needs above those of their siblings." (Female 60-69 years, White, Christian, DDKT)<br><br>"Both children have young children of their own who could possibly require a future transplant." (Male 60-69 years, White, Christian, DDKT)<br><br>"I would not allow my sons to take a risk on my behalf (all self-employed)." (Female 70-79 years, White, No religion, DDKT)<br><br>"The only person that could have donated a kidney to myself was my son. He was only in his twenties and just got married. I could not ask him and he did not offer." (Male, 60-69 years, White, DDKT)
